# Anti-Anaphylactic Activity of Isoquercitrin (Quercetin-3-O-β-d-Glucose) in the Cardiovascular System of Animals

**DOI:** 10.3390/biomedicines8060139

**Published:** 2020-05-29

**Authors:** Jinbong Park

**Affiliations:** Department of Pharmacology, College of Korean Medicine, Kyung Hee University, Seoul 02447, Korea; thejinbong@khu.ac.kr; Tel.: +82-2-961-2297

**Keywords:** isoquercitrin, cardiovascular anaphylaxis, rats, guinea pigs, histamine

## Abstract

Effects of isoquercitrin (IQ) on anaphylactic responses were examined in cardiovascular systems of experimental animals. In pithed rats, IQ at 30 and 100 mg/kg (intravenous) significantly blunted both the initial hypertensive and the ensuing hypotensive responses during anaphylaxis. Death rate and tachycardia were also significantly inhibited after the same IQ doses in these rats. In isolated guinea pig hearts, IQ infusion at 30–100 μg/mL markedly reduced anaphylaxis-related coronary flow decrease, contractile force change, and heart rate responses (both tachycardia and arrhythmia). Cardiac histamine and creatine kinase releases were similarly diminished by IQ during anaphylaxis in the isolated guinea pig hearts. In two different isolated guinea pig vasculatures, the pulmonary artery and mesenteric arterial bed, anaphylactic vasoconstriction was reduced by IQ 30 and 100 μg/mL. It was observed that IQ had a marked inhibitory effect on histamine release from rat mast cells, and this mechanism was suggested as the major anti-anaphylactic mechanism. Direct inhibition of histamine-induced muscle contraction did not seem to be relevant, but IQ treatment successfully repressed intracellular calcium influx/depletion in mast cells. Overall, this study provided evidence for the beneficial effect of IQ on cardiac anaphylaxis, thus suggesting its potential applications in the treatment and prevention of related diseases.

## 1. Introduction

Systemic anaphylaxis is a rare but a dramatic and fatal allergic disease with a prevalence of 0.05–2.0% in humans [1]. In anaphylaxis, cardiovascular systems play a critical role, both as a source and a target of various anaphylactoid mediators released during this highly life-threatening episode [2]. Heart functions fail as a result of arrhythmia and coronary vessel constriction, and the systemic blood vessels dilate, leading to worsened blood perfusion [3]. Appropriate pharmacological interventions can lead to an improved prevention and better recovery from anaphylactic symptoms, and natural substances can be good candidates to alleviate the damage arising from anaphylactic symptoms.

Flavonoids are plant-derived polyphenolic substances that are ubiquitously found in numerous herbal medicines, and quercetin is one of the most extensively studied substance of all flavonoids for its beneficial pharmacological activities [4]. However, quercetin (Q) occurs mostly as glycosides rather than in its aglycone form in nature. Typically found examples are quercitrin (Q-3-O-rhamnoside), rutin (Q-3-O-rutinoside), isoquercitrin (Q-3-O-glucoside), and hyperin (Q-3-O-galactoside).

Isoquercitrin (IQ) is frequently found in many plants including onions and numerous medicinal plants [5,6]. IQ was reported to have a variety of pharmacological actions, including anti-oxidant [7], anti-hypertensive [8], anti-cancer [9], and diuretic effects [10]. A few available studies hint the possibility that IQ could exert anti-anaphylactic activity because this glycoside inhibited certain biological processes known to be linked to allergic events [5,11].

In contrast to a wide range of reported beneficial activities of quercetin flavonoids, only limited information circumstantially suggests that IQ might be effective against allergic anaphylaxis. Furthermore, there are almost no studies on the preventive effect of flavonoids against cardiovascular anaphylaxis symptoms. In this study, the beneficial effect of IQ on ovalbumin (OVA)-induced cardiac anaphylaxis was evaluated to provide evidence for its use on allergic anaphylaxis.

## 2. Materials and Methods

### 2.1. Test Substance and Reagents

The test substance IQ was purchased from Sigma-Aldrich Inc. (St. Louis, MO, USA) (Product No. 00140585) and dissolved in the physiological buffers used in the in vitro experiments. For intravenous administration of IQ in pithed rats, IQ was dissolved in dimethylsulfoxide (DMSO) (Product No. 472301) and then injected slowly for about 1 min. DMSO (≥ 99.9%) was used for vehicle treatment. All other reagents were also purchased from Sigma-Aldrich Inc.

### 2.2. Experimental Animals

Rats (specific pathogen-free) and guinea pigs were purchased from Damul Science (Daejon City, Korea) and maintained in rodent chambers at 21 ± 1 °C and 55 ± 2% relative humidity. Feeds and drinking water were supplied ad libitum. All experimental protocols involving the use of animals conformed to the NIH guidelines (Guide for the Care and Use of Laboratory Animals, 8th edition). The Animal Care and Use Committee of the Institutional Review Board of Kyung Hee University approved all animal experiments (confirmation number: KHUASP(SE)-12-036, date: 1 May 2016).

### 2.3. Cardiovascular Anaphylaxis in Pithed Rats

Male Wistar rats (220–250 g) were actively sensitized with 10 mg/head of OVA (turkey OVA, Grade IV) (Product No. SAB4200702) injected intraperitoneally on day 1 and 2, once daily. On day 20, the rats were lightly anesthetized with ethyl ether and a tracheal cannula was placed for artificial respiration (Harvard Apparatus, Holliston, MA, USA), which ran at 60 strokes/min and 1 mL/100 g body weight rates. A pithing rod (round copper 12 cm long, 1.5 mm diameter) was inserted through the right orbit, the brain, and down to the sacral region in the spinal cord. During this process, the brain tissues were maximally destroyed [12]. The right common carotid artery and jugular vein were respectively cannulated with PE-50 cannulae for cardiovascular monitoring and intravenous drug administration. Test substance IQ (dissolved in DMSO) was bolus administered 10 min before intravenous OVA injection (1 mg/rat). Cardiovascular parameters were recorded with a physiography (Letica Polygraph 4006, Barcelona, Spain).

### 2.4. Anaphylaxis in Isolated Guinea Pig Hearts

Passively sensitized anaphylactic animal models were produced with male Hartley guinea pigs weighing 300–350 g [13]. Anti-OVA serum was administered to naïve guinea pigs, 24 hr before the experiments. The guinea pigs were lightly anesthetized with ethyl ether and the hearts were rapidly removed. An aortic cannula was placed and the heart was setup into a Langendorff heart apparatus for coronary artery perfusion [14]. The hearts were perfused with Krebs-Henseleit solution (in mM, NaCl 118, KCl 4.7, CaCl_2_ 2.5, MgSO_4_ 1.6, NaHCO_3_ 24.9. KH_2_PO_4_ 1.2, glucose 2.5, pH 7.4) under a constant pressure of 60 cm H_2_O at 37 °C. The perfusion solution was continuously saturated with 95% O_2_–5% CO_2_ gas. Cardiac contractility was continuously monitored with a TRI201 isometric transducer (Hugo-Sachs Electronik GmbH, March, Germany). Cardiac anaphylactic response was induced by delivering 1 mg of OVA into the perfusion buffer. When testing the effect of IQ, the IQ-containing buffer was infused to the heart for 10 min before anaphylaxis induction. Cardiac parameters were recorded with a physiography (Letica Polygraph 4006). Coronary effluent was collected at 1-min intervals for flow change monitoring and chemical analyses.

### 2.5. Anaphylaxis in Isolated Guinea Pig Mesenteric Arterial Beds

Male Hartley guinea pigs were passively sensitized in the same manner as in the isolated heart experiment. Under ether anesthesia, the animal was sacrificed by cervical exanguination and the mesenteric vascular bed was exposed through a midline incision on the abdomen. A stainless cannula was inserted into the superior mesenteric artery via the abdominal aorta. Blood remaining in the mesenteric arterial bed was disposed by perfusing with 20 mL Krebs–Henseleit buffer containing 2000 IU of heparin. The whole arterial bed was carefully isolated, keeping the surrounding arteriole-venule junctions intact [15]. The preparation was maintained in a 50-mL glass container at 37 °C, and continuously perfused with the Krebs-Henseleit buffer (saturated with 95% O_2_–5% CO_2_, pH 7.4) at 5 mL/min rate. Perfusion pressure was monitored with a pressure transducer (Letica). IQ was infused into the preparation 10 min prior to the OVA (1 mg) challenge.

### 2.6. Anaphylaxis in Isolated Guinea Pig Pulmonary Artery

From the same guinea pigs that were used for the mesenteric arterial beds, the pulmonary arteries were isolated. Excised artery was cut into ring segments of 2–3 mm. The segments were suspended using two stainless stirrups in a water jacked 10-mL organ bath maintained at 37 °C. Rings were submerged in Krebs–Henseleit buffer (pH 7.4) saturated with 95% O_2_–5% CO_2_. Constant tension of 1.0 g was applied to the rings and a TRI201 isometric transducer was connected for tension measurements. OVA (1 mg) was applied to the bath to elicit anaphylactic contraction. IQ was exposed to the artery, 10 min prior to anaphylaxis.

### 2.7. Histamine Release in Rat Peritoneal Mast Cells

Naïve male Wistar rats (250–300 g) were injected with 20 mL of phosphate-buffered saline (mM, NaCl 137, KCl 2.7, CaCl_2_ 1.8, MgCl_2_ 1.1, NaH_2_PO_4_ 0.4, NaHCO_3_ 11.9, glucose 5.5, HEPES 1.0, pH 7.4). The abdomen was gently massaged for a few minutes and the abdominal fluid was obtained. Mast cells were isolated by centrifugation of the fluid (200× *g*, 5 min) and suspended at 1 × 10^5^ cells/mL, following Percoll density gradient method (Erenbeck and Svensson, 1980). The purity of the mast cells was ≥97%, when determined by a toluidine blue staining. After stabilization at 37 °C, histamine release was evoked by adding either 0.5 μM of compound 48/80 (Product No. C2313) or 1 μM of calcium ionophore A23187 (Product No. C7522). Releases were terminated by freezing the cells following 15-min incubation, in the presence or absence of IQ.

### 2.8. Antagonism of IQ Against Histamine in Isolated Guinea Pig Guinea Trachea and Ileum

Naïve male Hartley guinea pigs (350–380 g) were used to examine direct effects of IQ on histamine-induced muscle contractions. The tracheal strip was composed of two rings excised with 3 mm width, opened, and sutured together with a silk thread. The preparation was suspended in a 15-mL organ bath maintained at 37 °C [15]. Ring strips were submerged in Krebs–Henseleit buffer (saturated with 95% O_2_–% CO_2_, pH 7.4) and a resting tension of 500 mg was applied. Distal portion of the ileum was cut at 1.5 cm long and mounted in a 20-mL organ bath kept in the Tyrode’s solution at 37 °C [16]. The solution was saturated with 95% O_2_–5% CO_2_ and 1 g resting tension was applied. Both muscles were exposed to IQ for 10 min, prior to histamine (1 μM) addition. Tension changes were measured with a TRI201 isometric transducer and strip chart recorder.

### 2.9. Analysis of Histamine Concentration and Creatine Kinase Activity

Histamine concentrations in the buffers of heart perfusion and mast cell incubation were performed [17]. The guinea pig heart perfusate was used directly for the histamine measurements. However, the mast cell culture was centrifuged (2000× *g*, 5 min, 3 °C) to obtain the medium for the released histamine. Mast cell pellets were boiled for 5 min and then used for intracellular histamine measurements. Released histamine was conjugated with o-phthalaldehyde (Product No. P1378) to produce a fluorescent product, and the fluorescence signals were measured with a fluorometer, at 360 nm excitation wavelengths and 450 nm emission wavelengths. The ratios were calculated from the two values. Creatine kinase activity in the guinea pig heart effluent was assessed using creatine phosphokinase from the rabbit muscle (Product No. C3755), as previously described [15].

### 2.10. Intracellular Calcium Level Measurement

The intracellular calcium was measured with the use of the fluorescence indicator Fura 2-AM (Product No. F0888). HMC-1 cells (1 × 10^5^ cells) were pre-incubated with Fura 2-AM for 45 min at 37 °C. After being washed to remove the remaining Fura 2-AM, HMC-1 cells were treated with IQ (100 μg/mL) or cromolyn sodium (100 μM) (Product No. 1150502) for 20 min. Intracellular calcium depletion was measured by stimulation with 20 nM of phorbol 12-myristate 13-acetate (PMA) (Product No. P1585) and 1 μM of calcium ionophore A23187 treatment. The intracellular calcium influx was measured by applying calcium in the cell media. The signals were measured with excitation wavelengths of 340 and 380 nm, by a spectrofluorometer FluoroMax^®^-3 (Horiba Ltd., Kyoto, Japan).

### 2.11. Statistical Analysis

Data were expressed as mean ± standard error. Statistical significance between groups was analyzed with one-way analysis of variance, followed by Newman-Keul’s *t*-test. *p*-values of 0.05 were used for the significance criteria.

## 3. Results

### 3.1. IQ Alleviates Cardiovascular Anaphylaxis in Pithed Rats

**Figure 1:**
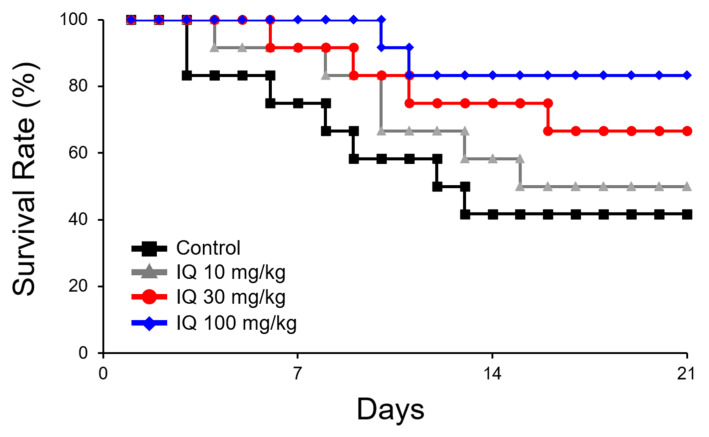
Effect of isoquercitrin (IQ) on mortality caused by cardiovascular anaphylaxis in pithed rats. Male Wistar rats (*n* = 12 per group) were intraperitoneally sensitized with 10 mg/head of ovalbumin (OVA) on day 1 and 2. Survival rate was observed for 20 days. DMSO was used as the control.

**Figure 2:**
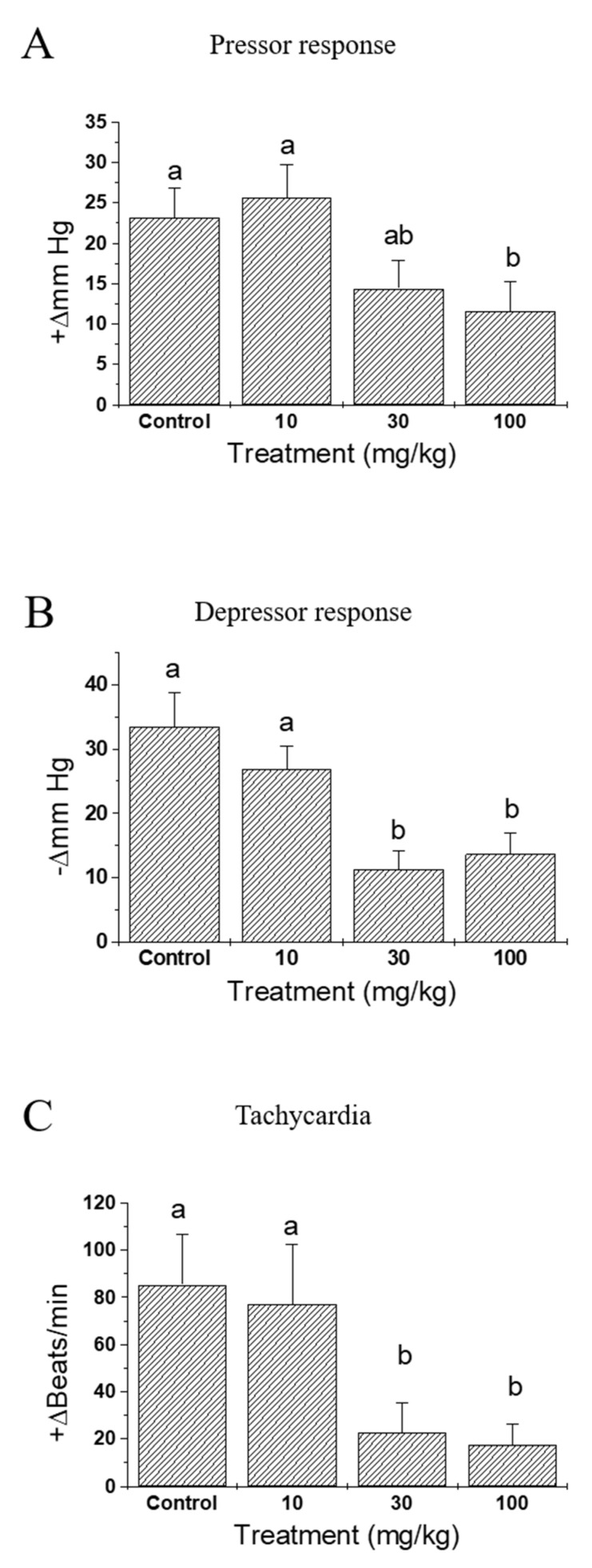
Effects of isoquercitrin (IQ) on cardiovascular anaphylaxis in the pithed rats. Male Wistar rats (*n* = 12 per group) were intraperitoneally sensitized with 10 mg/head of OVA on day 1 and 2. (**A**) Pressor response, (**B**) depressor response, and (**C**) the tachycardia rate were measured in the cardiovascular system of the pithed rats. DMSO was used as control. Different alphabets on the bars denote significantly different means at *p* < 0.05.

By pre-treatment with IQ, a dose-related reduction in mortality was noted (Figure 1). A drastic 3.5-fold decrease was observed in 100 mg/kg of IQ when compared to the vehicle-treated control rats. Figure 2 illustrates the effects of IQ on OVA-induced cardiovascular anaphylaxis in sensitized and pithed rats. Administration of 30 and 100 mg/kg IQ, prior to the OVA challenge reduced all functional cardiovascular changes, including the pressor response (Figure 2A), depressor response (Figure 2B), and tachycardia (Figure 2C). However, 10 mg/kg dose did not influence the parameters.

### 3.2. IQ Improves Cardiac Anaphylaxis in Isolated Guinea Pig Heart

**Figure 3:**
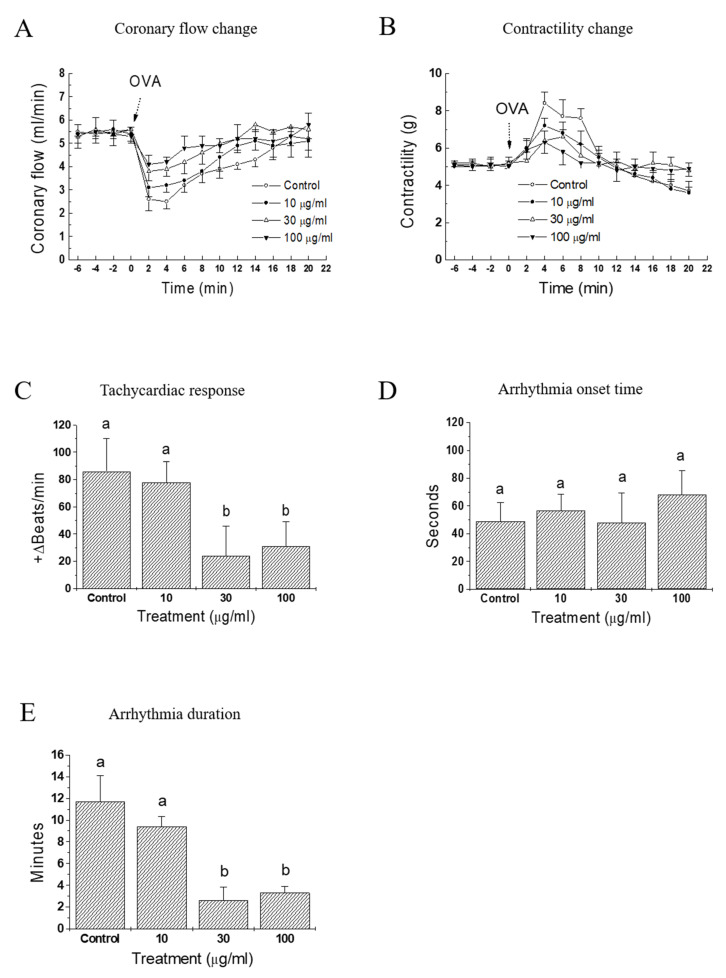
Effects of isoquercitrin (IQ) on cardiac anaphylaxis in isolated guinea pig hearts. (**A**) Coronary flow change, (**B**) contractility change, (**C**) tachycardiac response, (**D**) onset, and (**E**) duration time of arrhythmia were measured in OVA (1 mg)-treated isolated hearts from Hartley guinea pigs (*n* = 10 per group). DMSO was used as the control. Different alphabets on bars denote significantly different means at *p* < 0.05. Statistical significance was not shown for clarity purpose in the coronary flow change and contractility change.

As in vivo anti-anaphylactic effects were observed in rats, it was examined whether a similar protective activity was also present at the heart levels. OVA-treated guinea pig heart was selected as an ex vivo approach to evaluate the effect of IQ. In response to OVA, heart rate started to increase (tachycardia) markedly but lasted briefly (< 1 min), then it turned into an irregular rate (arrhythmia), which rarely disappeared during the 20-min observation period (data not shown). Infusion of the test substance IQ before the OVA challenge blunted these anaphylactic responses, i.e., coronary flow, contractility, and tachycardia in a concentration-dependent manner (Figure 3A–C). However, the lowest concentration (IQ 10 μg/mL) was ineffective in most parameters except on contractility changes. In the coronary flow, IQ 10 μg/mL was temporally effective on the recovery phase of flow (8–16 min post-OVA, Figure 3B). Arrhythmia onset time was not delayed by IQ (Figure 3D), however its duration was remarkably reduced by 30 and 100 μg/mL of IQ pre-treatment (Figure 3E).

### 3.3. IQ Ameliorates Anaphylaxis in Isolated Vasculatures of Guinea Pig Heart

**Figure 4:**
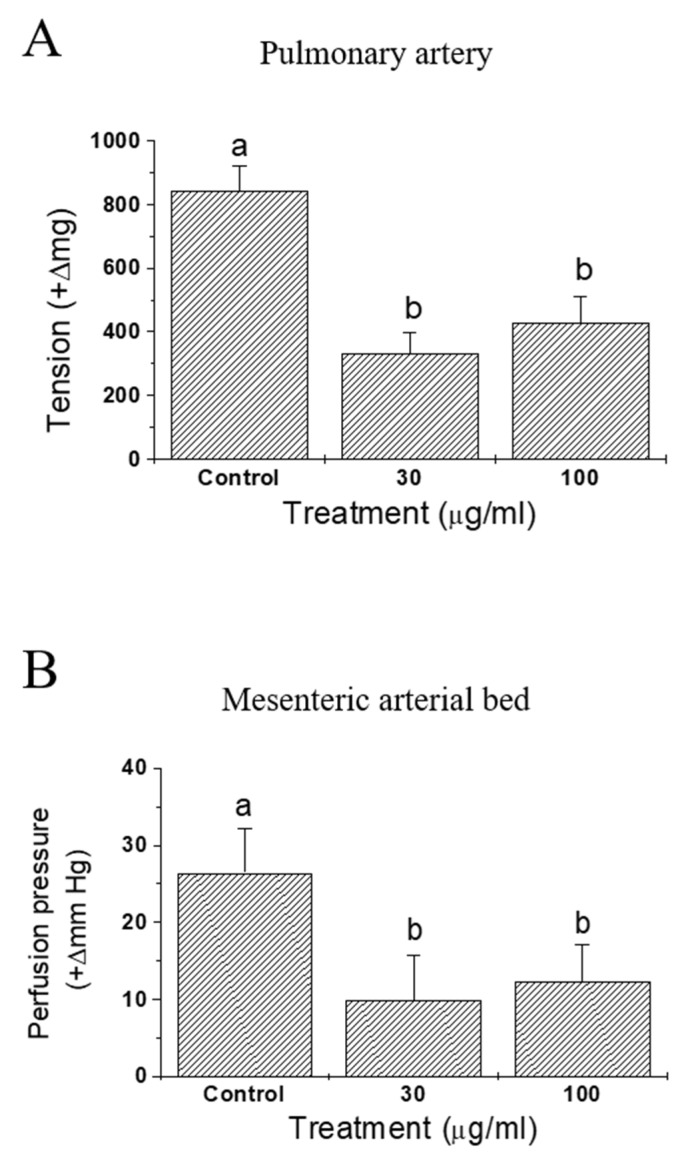
Effects of isoquercitrin (IQ) on anaphylactic responses in isolated guinea pig blood vessels. (**A**) Pulmonary artery tension and (**B**) pressure in mesenteric arterial bed were measured in isolated blood vessels from the Hartley guinea pigs (*n* = 7 per group). DMSO was used as the control. Different alphabets on the bars denote significantly different means at *p* < 0.05.

Effects of IQ on anaphylactic responses were examined in sensitized isolated guinea pulmonary artery and mesenteric arterial beds (Figure 4). In both preparations, vasoconstrictive responses were monitored and the test substance IQ was effective in significantly blocking OVA-induced vasoconstriction—tension increase in pulmonary artery (Figure 4A) and perfusion pressure increase in mesenteric arterial beds (Figure 4B).

### 3.4. IQ Suppresses Histamine and Creatinine Release in Isolated Guinea Pig Heart and Rat Mast Cells

**Figure 5:**
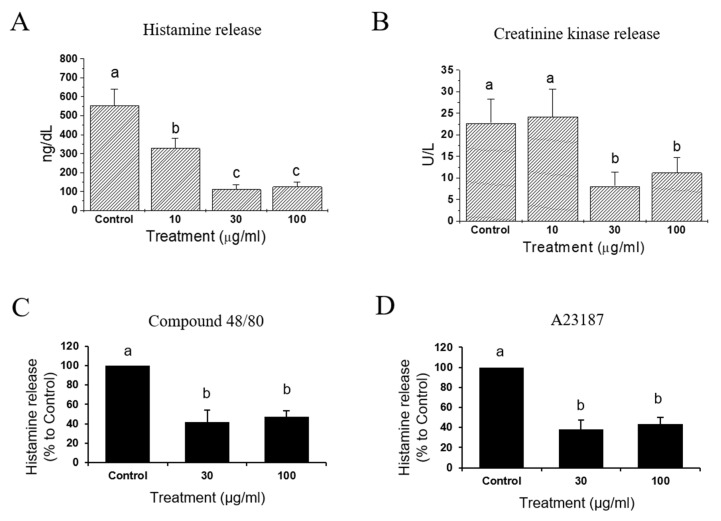
Effects of isoquercitrin (IQ) on histamine and creatinine kinase release. (**A**) Histamine and (**B**) creatinine kinase release were measured in OVA (1 mg)-treated isolated hearts from Hartley guinea pigs (*n* = 10 per group). Basal histamine and creatine kinase levels were 0–52 ng/dL and 0 IU/L during the resting and the pre-challenge periods, respectively. (**C**) Compound 48/80 (0.5 μM)-induced and (**D**) A23187 (1 μM)-induced histamine release in rat peritoneal mast cells were measured (*n* = 7 per group). DMSO was used as control. Different alphabets on the bars denoted significantly different means at *p* < 0.05.

Concomitant with the functional changes that occurred during the anaphylactic event, histamine and creatine kinase levels markedly increased over their baselines. These elevations were also bunted by IQ in a concentration-dependent manner. Effects on creatine kinase activity by IQ was an indication that IQ possessed preventive activities against the myocardial damage occurring in anaphylaxis. As histamine concentration in the coronary effluent decreased by IQ in the isolated heart model, effects of IQ on histamine release was directly examined in the purified rat mast cells. Compound 48/80 (Figure 5C) or ionophore 23187 (Figure 5D) elicited histamine release and this release was significantly reduced by IQ at 30 and 100 μg/mL (>50% inhibition).

### 3.5. IQ Did not Affect Direct Muscle Contraction by Histamine in Tissues Isolated from Guinea Pig

**Figure 6:**
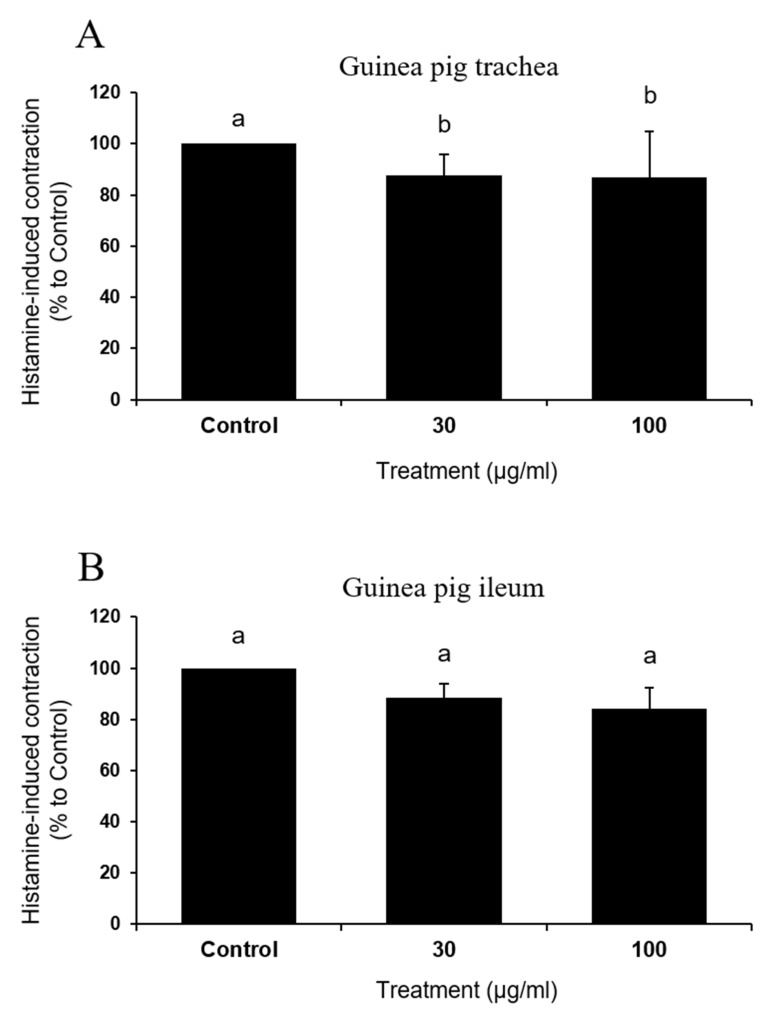
Effects of isoquercitrin (IQ) on histamine-induced muscle contractions. Histamine (1 μM)-induced muscle contraction in (**A**) trachea and (**B**) ileum tissues were measured (*n* = 5 per group). DMSO was used as the control. Different alphabets on the bars denote significantly different means at *p* < 0.05.

To examine if the test compound exerted direct inhibition on histamine, effects of IQ on histamine-induced contraction were assessed in the trachea and ileum of guinea pigs (Figure 6A and B). Surprisingly, IQ was not effective or were only marginally effective (significant inhibition with 100 μg/mL in trachea, *p* = 0.04) in influencing contractile responses in both of these muscle preparations.

### 3.6. IQ Stabilizes Mast Cells and Inhibits OVA-Stimulated Intracellular Calcium Release

**Figure 7:**
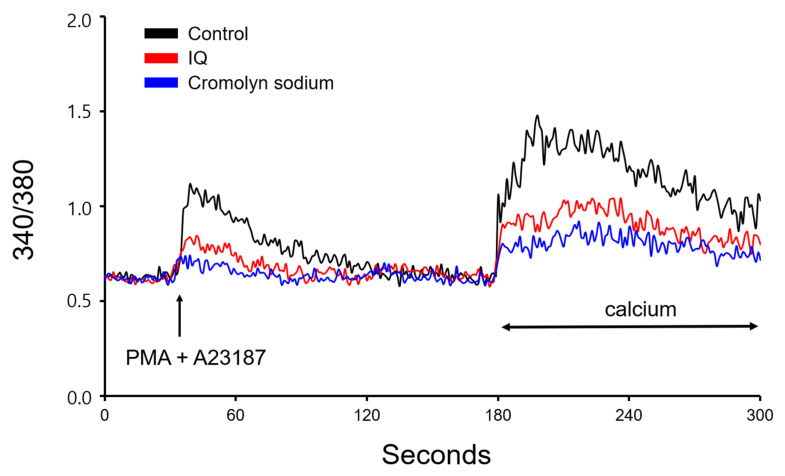
Effects of isoquercitrin (IQ) on PMA + A23187-induced intracellular calcium levels in HMC-1 cells. Intracellular calcium levels were measured for 300 s. PMA (20 nM) and A23187 (1 μM) was used to stimulate the HMC-1 cells. DMSO was used as a control.

Since IQ did not protect muscles from histamine stimulation but did inhibit histamine release, we next evaluated its effect on intracellular calcium levels. As shown in Figure 7, positive control cromolyn sodium inhibited calcium depletion caused by PMA + A23187 in HMC-1 cells. Similar results were observed by pre-treatment with IQ, and in addition, both cromolyn sodium and IQ suppressed calcium influx in mast cells as well.

## 4. Discussion

Pithed rats in the anaphylaxis study showed an advantage in the assessment of the overall cardiovascular changes under silent conditions, without the participation of compensatory reflex adjustments [12]. Anaphylactic responses in this in vivo animal model were characterized as a brief increase in blood pressure (pressor response), followed by a gradual decline (depressor response) that frequently led to death [12,15]. Heart rates increased (tachycardia) markedly and sustained at the elevated levels for several minutes. When IQ was treated at 10, 30, and 100 mg/kg, the treatment did not only decrease the overall mortality (Figure 1), but also alleviated pressure/depressor response and tachycardia in pithed rats (Figure 2), suggesting a dose-dependent improvement on cardiac anaphylaxis.

When the antigen OVA was injected into pre-sensitized, isolated guinea pig hearts, immediate and dramatic cardiac functional changes occurred [18]. Namely, the coronary flow diminished markedly and the contractile force increased initially (in about 2 min post-OVA challenge in this study), then both of these changes gradually returned to pre-challenge values. IQ pretreatment suppressed OVA-induced anaphylactic responses, including coronary flow, contractility, tachycardia, and duration of arrhythmia (Figure 3). These results clearly suggest the beneficial intervention of IQ during OVA-induced cardiac anaphylaxis. Moreover, when sensitized isolated guinea pulmonary artery and mesenteric arterial beds were used in the same manner as that of OVA treatment, IQ significantly reduced the tension or perfusion pressure in these two isolated vasculatures (Figure 4). These data had a meaning that it was possible to confirm the anti-anaphylactic activity of IQ at isolated blood vessel levels—the isolated pulmonary artery as a representative large vessel and the mesenteric arterial bed as a capillary blood vessel.

Dramatic functional changes in cardiac anaphylaxis were well-described [19] and such reactions reflected the biological actions of endogenous mediators released from cardiac tissues [20]. By IQ treatment of 10, 30, and 100 μg/mL in isolated guinea pig hearts and 30 and 100 μg/mL in primary cultured rat mast cells, histamine release by OVA (guinea pig heart) or Compound 48/80 and A23187 (rat mast cell) were significantly suppressed (Figure 5). While the release of histamine, an important anaphylactic mediator [19], was reduced by IQ at 10 μg/mL, this concentration failed to reduce creatine kinase output in the coronary effluent. Such discrepancy might be explained by the fact that histamine is not the only anaphylactic mediator in hearts [3]. However, in cardiovascular anaphylaxis, histamine is known to play the most important role, although other mast cell-derived mediators such as leukotrienes, platelet-activating factor, thromboxane A_2_, and 5-hydroxytryptamine are also involved [21,22]. Thus, it is not unreasonable to assume that IQ could inhibit the release of other mediators in addition to histamine. Further study must evaluate the changes in such mediators in order to verify the specific action mechanism of IQ besides histamine. Notably, IQ did not affect the histamine-induced changes in guinea pig trachea/ileum (Figure 6), implying that most part of the anti-anaphylactic activities can be ascribed to inhibition of mediator release. IQ is known to directly antagonize leukotriene D_4_- and carbachol-induced tracheal muscle contractions but not those induced by histamine [5].

Intracellular calcium is known to be the most important pathway of histamine release in mast cells [23]. Thus, mast cell stabilizers such as cromolyn sodium or nedocromil [24] work by blocking a calcium channel that is essential for the degranulation of mast cells. Since IQ treatment showed beneficial effects in cardiac anaphylaxis but could not repress muscle contraction through direct histamine treatment, another logical approach might be to check its effect on mast cell stabilization. The results in Figure 7 indicate that IQ exerts such effect at a similar level to cromolyn sodium, a widely used mast cell stabilizer [25]. Though a direct comparison on histamine-related anaphylaxis was not provided, the data suggests that IQ possesses the potential efficacy as a conventionally used medication for allergic responses. In addition, confirming the effect of IQ along with several anti-anaphylactic agents (adrenalines, H1 antihistamine, glucocorticosteroid, etc.) [26] would provide important information and might justify the application of IQ.

This study demonstrated that IQ is active against cardiovascular anaphylaxis not only in the whole animal, but also in isolated hearts or vessels. However, this study indeed had its own limitations. The action mechanism of IQ seemed to be the inhibition of histamine, the most important anaphylactic mediator, from mast cells although additional minor mechanism(s) are still unknown. There are some reports that suggest the possibility that quercetin or quercetin glycosides could be anti-allergic [27,28,29]. While most biological activities are attributable to the aglycone form quercetin, its glycoside IQ was more potent than quercetin in some pharmacological effects [6,30]. Yet, because there are several pathways in the upstream channel of histamine release, such as the Fc epsilon receptor pathway or IgE and interleukins [31: Respir Med. 2012 Jan; 106(1):9–14.], extensive investigation regarding the action mechanism of IQ must be carried out. Whether IQ regulated these signaling pathways was not fully elucidated in this study but rather proved the sole fact that IQ suppressed histamine release from mast cells and thus improved cardiac anaphylaxis. More importantly, effect on the mast cell-derived mediators that drive allergic reactions after degranulation, besides histamine, should be confirmed as well, before being accepted as an anti-anaphylaxis agent, at least provisionally.

In summary, as cardiovascular anaphylaxis is a highly detrimental allergic episode that could result in fatal consequences in humans, exploring phytochemical candidates against this disease would be meaningful. Being ubiquitously present in nature and widely consumed through diets, IQ deserves further investigation for invention of a preventive measure against cardiovascular anaphylaxis.
